# Palliative care in the pre-hospital service in Brazil: experiences of health professionals

**DOI:** 10.1186/s12904-021-00890-4

**Published:** 2022-01-04

**Authors:** Jacqueline Resende Boaventura, Juliana Dias Reis Pessalacia, Aridiane Alves Ribeiro, Fabiana Bolela de Souza, Priscila Kelly da Silva Neto, Maristela Rodrigues Marinho

**Affiliations:** 1 Federal University of Mato Grosso do Sul (UFMS), Av. Ranulpho Marques Leal, n° 3484, Três Lagoas, MS Caixa-postal: 210 Brazil; 2 Federal University of Goiás (UFG)Federal University of Jataí (UFJ), BR 364, km 195, n° 3800, Jataí, GO Brazil; 3 University of São Paulo, Ribeirão Preto School of Nursing (USP), Avenida dos Bandeirantes, 3900 - Campus Universitário - Bairro Monte Alegre, Ribeirão Preto, SP Brazil

**Keywords:** Palliative care, Emergency medicine, Emergency professionals, Ambulances

## Abstract

**Background:**

An integrated care network between emergency, specialized and primary care services can prevent repeated hospitalizations and the institutionalized death of terminally ill patients in palliative care (PC). To identify the perception of health professionals regarding the concept of PC and their care experiences with this type of patient in a pre-hospital care (PHC) service in Brazil.

**Methods:**

Study with a qualitative approach, of interpretative nature, based on the perspective of Ricoeur’s Dialectical Hermeneutics.

**Results:**

Three central themes emerged out of the professionals’ speeches: (1) unpreparedness of the team, (2) decision making, and (3) dysthanasia.

**Conclusions:**

It is necessary to invest in professional training associated with PC in the home context and its principles, such as: affirming life and considering death as a normal process not rushing or postponing death; integrating the psychological and spiritual aspects of patient and family care, including grief counseling and improved quality of life, adopting a specific policy for PC that involves all levels of care, including PHC, and adopt a unified information system, as well as more effective procedures that favor the respect for the patients’ will, without generating dissatisfaction to the team and the family.

## Background

The World Health Organization (WHO) brings as a definition for Palliative Care (PC) “assistance promoted by a multidisciplinary team, which aims to improve the quality of life of patients and their families, in the face of a life-threatening disease, through prevention and relief of suffering, early identification, impeccable evaluation and treatment of pain and other physical, social, psychological and spiritual symptoms”. Moreover, it recommends the beginning of PC at the moment of the diagnosis of the disease, and not only at the end of life, recommending the integration between services at all levels of care, focusing on the targeting of Primary Health Care (PHC) [1].

The International Association for Hospice & Palliative Care (IAHPC) proposed and developed a project for the adoption of a new definition for PC regarding what it is, when it should be applied, to whom and by whom, encompassing all dimensions of life. It suggests the offer of holistic and active care to all people who experience intense suffering, resulting from diseases with no possibility of cure, especially those at the end of life. It also aims to improve the quality of life of patients, family members and caregivers who face issues associated with chronic diseases with life risk, through the prevention and relief of suffering [1, 2].

Accordingly, the guiding principles of PC are: to affirm life and consider death as a normal process; not to rush or postpone death; to integrate the psychological and spiritual aspects of patient care; to offer a support system to help patients live as actively as possible until death; to offer a support system to help the family deal with the patient’s illness and in their own grief; to use a team approach to meet the needs of patients and their families, including bereavement counseling if indicated; improve quality of life and also positively influence the course of the disease; is applicable at the beginning of the course of the disease, in conjunction with other life-long therapies such as chemotherapy or radiotherapy, and includes the investigations needed to better understand and manage distressing clinical complications [3].

The PC offer covers a wide variety of settings including hospitals, hospices, nursing homes and domiciles and such care is generally classified when it is performed by general practitioners, who play a key role in an integrated PC model, applying their knowledge and skills to care for the patient. These professionals are aware of the formal and informal health and social services available in the community, making it possible to refer, when necessary, to the services that can assist in the care or carried out by specialized professionals [4], which are offered by hospitals, which promote patient and family care and/or coordination and information for other specialized health services, hospices and home care, to help them provide support to the patient and family [5].

In Brazil, the PC offer is still focused on hospitals and, even so, only 10% of these institutions provide a specialized team [6]. Other authors [7] argue that the integration of PC in the PHC may solve the absence of PC specialists, becoming a tangible starting point in the transformation of the current reality. In Brazil, Emergency Services (ES) are public and linked to the Urgency and Emergency Network (UEN), organized and regulated under the Unified Health System (*SUS-SistemaÚnico de Saúde*) by Ordinance No.1.600 of 2011. Among the components of this network is the Mobile Emergency Care Service (SAMU), which began its activities in 2003 and has been expanding throughout the country ever since [8].

The Pre-Hospital Care (PHC) had a military origin in the XIX [9] century, which had the need to rescue immediately and save wounded soldiers in the fighting. The military influence was also present in the Brazilian context, as the Rio de Janeiro Fire Department was the first to provide services outside the hospital environment. The PHC model adopted in Brazil was based on the French and American examples, centralized in a communications network, and based on medical regulation and the emergency call through a single telephone number [9].

Currently, the SAMU is the main mobile component of the Health Care Network (HCN) in PHC in Brazil, is linked to the SUS and serves 75% of the population through ambulances. The role of SAMU is to attend to more complex cases, such as severe trauma and cardiorespiratory arrest, where severity exposes the patient to increased risk of death on the place [10] and thus decrease hospital admissions, deaths, and sequel related to waiting time [11].

However, the care given to terminally ill patients is a challenge by the teams working in ambulances in the emergency service [12]. Considering the care of patients eligible for PC, the role of urgency and emergency services, according to Brazilian guidelines for the organization of PC within the SUS, is to provide care to relieve acute symptoms, focused on comfort and dignity of the person, following the best practices and available evidence [13].

However, the SAMU can represent an important link with specialized services in this care, improving the patient’s quality of life, avoiding unwanted hospital admissions and medical expenses [14]. Also, through calls for assistance to palliative patients in cardiac arrest, they play a role in patient evaluation, the transmission of bad news, and support to family and passersby.

PCs become exclusive during the progression of chronic diseases, but the final stage of the disease is often related to moments of agony and suffering. Thus, the death of a patient in PC can happen in services where the care of terminally ill patients does not coincide with the usual routine exercised by professionals. For example, ES, which aims to stabilize and preserve the lives of people in acute events [15]. Due to the understanding of these professionals that the role of the service is to treat acute symptoms and stabilize the patient, they resort to extraordinary measures in patients eligible for PC, such as cardiopulmonary resuscitation (CPR) and other invasive and futile procedures. In Brazil, Non-resuscitation Orders (NRO) are not supported by legislation, which requires health professionals to apply CPR in all cases, except when death is unquestionable [16], causing an increase in hospitalizations and prolongation of the patient’s suffering and their family members.

Effective communication between the services of the integrated care network, involving an emergency, specialized service, and primary care professionals could minimize the dilemmas related to these behaviors, as the PHC professionals could have access to the patient’s previous medical history, preventing futile procedures, repeated hospitalizations and institutionalized deaths of terminally ill patients [17].

However, in addition to the communication barrier, patients, family members, and health professionals have difficulties in interpreting the concept of PC and its practical implementation. Therefore, they manifest difficulties in selecting patients who would benefit from PC, which may lead to delays in meeting the needs of the patients [18].

Due to the Brazilian context of the discussion of a policy for PC integrated into the SUS health care network, however, in the home environment, in which the patient and family do not have guidance on how to proceed in an emergency, and also regarding the lack of clarity of health professionals on the concepts of PC and the roles of the PHC service in this type of care in Brazil, this study aimed to identify the perception of health professionals regarding the concept of PC and their care experiences, observing the limits and possibilities, professional preparation, also the outcome of the care provided with this type of patient in a PHC service, in Brazil.

We are based on the hypothesis that PHC service professionals in Brazil do not understand the concept of PC and the roles of the mobile service in caring for this type of patient and that their lack of preparation is related to the implementation of futile and which contribute to the increase in unnecessary hospitalizations and the suffering of the patient and his family. We also consider that the lack of effective communication between the services that make up the care network in the country amplifies ethical conflicts in the care provided by PHC professionals and hinders access to quality care for patients and their families.

## Methods

Study with a qualitative approach, of interpretative nature [19], based on the perspective of Ricoeur’s Dialectical Hermeneutics [20, 21].

We sought to understand social reality through the intersubjectivities of human relations and their interactions with the environment. Relations and representations are considered a fundamental part of the successes and limits of actions [22].

The use of hermeneutics seeks the meaning of communication between human beings, through the interlocution of daily life and common sense, focusing on language as the central nucleus. Dialectics, on the other hand, presents itself as the art of dialogue, of questioning and controversy, searching in facts, in language, in symbols and in culture, the obscure and contradictory nuclei. Thus, such methodology seeks to understand the point of view of thoughts through the synthesis of understanding and critical processes [23].

### Setting and sample

This research was carried out between August and September of 2017, at the city’s SAMU base, and the free service is offered to the entire population, which operates 24 h a day, by means of guidance services and sending ambulances, manned by teams of properly trained professionals, which are activated by a Central Emergency Regulation Center.

The choice of location was guided by the following criteria: high prevalence of PC services by the team and no previous evaluation on PC in the service. In 2017, this PHC service attended a total of 354 occurrences to patients eligible for PC according to the WHO criteria. Such criteria consider for eligibility that the patient presents one or more of the following pathologies or conditions: Alzheimer’s disease and other dementias, cancer, cardiovascular disease (excluding cause of sudden death), cirrhosis of the liver, congenital anomalies, meningitis, hematological and immunological diseases, neonatal conditions, chronic obstructive pulmonary disease (COPD), diabetes, acquired human immunodeficiency syndrome (HIV/AIDS), renal failure, multiple sclerosis, Parkinson’s disease, rheumatoid arthritis and drug-resistant tuberculosis [1].

Nevertheless, one of the most accepted eligibility criteria for PC refers to the patient’s lifetime, where Medicare (a health insurance system managed by the U.S. government) establishes a six-month life expectancy period for exclusive PC referral. In order to establish this prognosis and indication of PC, the use of the ‘capacity for daily life activities’ construct is recommended. Based on this concept, the indication of PC becomes necessary through the inability of these patients to perform activities such as locomotion, feeding and also incontinence, for which some scales of assessment are proposed [24].

The study comprised the 25 health professionals working in the SAMU intervention in a municipality in the state of Mato Grosso do Sul, central region of Brazil. The professional categories were medicine, nursing, technical level (middle level qualification with the aim of training the student with theoretical and practical knowledge in that area) and higher level, drivers/first responders. For inclusion of the participants, we considered the experience of 2 years or more in PHC and age over 18 years old. We excluded professionals who were on sick leave and/or vacation during the interviews.

### Data collection

The team responsible for data collection was composed of two nurses (JRB, welfare nurse of SAMU and JDRP, teaching nurse), which was previously trained by the research supervisor. There was previous contact with the professionals in order to invite them to participate in the research and, after acceptance, the day and time were scheduled for the consent process regarding ethical issues and data collection, which occurred on the same day at the workplace, in a private room.

The authors used a semi-structured interview, composed of questions related to the experience of the professionals with patients eligible for PC during the SAMU consultations. The elaboration of the questions for the script considered the professional experience of the researchers in ES, the theoretical framework and objectives of the research, the information that the researchers compiled about the social phenomenon 30.

From this, the interview script was elaborated considering the studied reality, in order to include terms and expressions common to the participants. This aspect allowed for alignment and understanding by the respondents about the objective of each question. The script was then applied with a professional from another PHC service to validate the content and clarity of the script.

As the data analysis followed Ricouer’s Dialectic Hermeneutics, the collection and analysis were performed concomitantly. This fact provided the improvement of the questions in order to deepen the understanding of the object of study [21], as well as to adjust the level of understanding of each question by the participant.

Thus, the interview conducted considered the following guiding questions: (1) What do you understand about Palliative Care? What are the limitations and possibilities that you find when providing this type of assistance in the emergency service? (2) In emergency services, professionals seek to save lives. Do you think you are prepared to ‘deal’ with the death of a terminally ill patient during a call in which the SAMU is requested to promote comfort and/or pain relief at that moment? Why? (3) What is your perception about the outcome of the care provided by SAMU that impacts on the quality of life of the palliative patient?

However, the questions served as a means of facilitating the conversation in order to encourage participants to dialogue their experiences and perspectives as they wished and ask additional questions that arose during the dialogue. As is typical of qualitative interpretive description data collection, we did not strictly follow the interview scripts, but instead the interviewers followed the participant’s orientation, encouraging the elaboration of aspects of their experiences related to the care of patients in PC.

The interviews with the professionals working in the ES were held in a private room within SAMU itself, according to the participant’s preference, and lasted between 45 and 120 min. All interviews in the study were recorded on audio and transcribed in full, it is worth noting that only minimal demographic information was collected from the participants, as it is an environment with approximately 60 professionals, in order to ensure anonymity.

The delimitation of the number of participants took place from the moment the data collected responded to the objectives of the study. That is, new participants were not recruited at the time when the interviews conducted provided sufficient data to understand the overall picture and depth of the study object.

### Ethical aspects

Data collection started after approval by the Research Ethics Committee of the Federal University of Mato Grosso do Sul (UFMS), CAAE 98491218.2.0000.0021, under opinion n° 2,921,437. Consent was obtained in a two-stage process, according to Resolution 466/2012 of the National Health Council/Ministry of Health (NHC/MOH). Before starting each interview, the participants were verbally informed about the objectives, risks and benefits, and methods to be employed in the study.

Subsequently, the signatures of the TCLE were obtained, prepared in two copies, one of which was signed by the researchers and delivered to the participant and the other was signed by the participant and returned to the researchers. The interviews after transcribed were identified by ordinal numbers randomly to ensure secrecy and anonymity of participants.

### Data analysis

The data collection and analysis were guided by the Ricoeur’s Dialectic Hermeneutics [20]. As each interview was conducted, its recording was transcribed and the thematic analysis began with the initial codification of the material. For this purpose, the team proceeded to an exhaustive reading of the totality of the interviews, in order to carry out the hermeneutical movement of the whole to the parts and vice-versa [20, 21]. The process of translation into English occurred after the manuscript was finished, before being sent to the journal.

The data analysis followed the steps: I) Reading of the material (records of observations, transcribed interviews, and institutional documents); II) Extraction of information related to the study; III) Definition of pre-categories according to the research objectives and theoretical framework; IV) Inference, and after identification of the main meanings, transformation of the pre-categories into thematic categories [19].

## Results

Twenty-five interventionist professionals who work in PHC ambulances responded to the interview. The studied social group comprised seven physicians, four nurses, six nursing technicians and eight drivers/rescuers. In this universe, 13 professionals had up to 3 years of experience in PHC, three had from four to 7 years, and nine had more than 7 years of experience in the emergency service.

The consideration of all the speeches showed a transversal understanding of PC and of the assistance provided by the PHC team. Therefore, central and interdependent themes were obtained from the analysis of the data corpus, from the perspective of the dialectical hermeneutic movement [20], namely: unpreparedness of the team, decision making and dysthanasia. These themes converge into challenges for the care to PC patients.

**Fig. 1:**
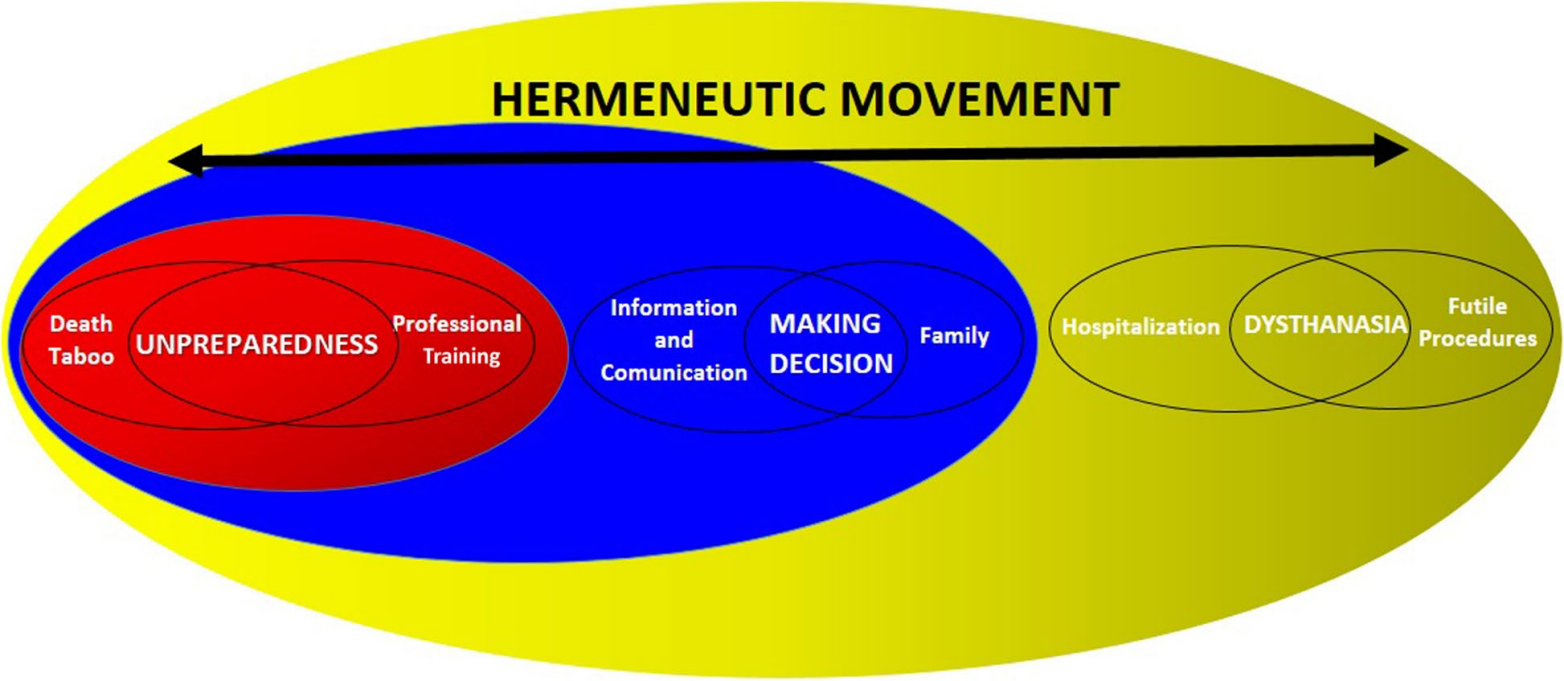
Central themes and their respective nuclei of meaning about the professionals’ experiences and the care provided in cases of patients eligible for PC. Três Lagoas (MS), Brazil, 2020

Figure 1 summarizes the themes and their respective nuclei of meaning emerging from the conclusion of the hermeneutical movement of analysis of the interviews, considering the experiences of professionals and the care provided to patients eligible for PC.

### Theme: unpreparedness of the team

The theme ‘unpreparedness’ represents the perceptions of PHC professionals regarding the unpreparedness of health professionals to deal with death and to provide care for patients in PC. The core meanings of this theme are: Death as a taboo and Professional training.

### Subtopic: death as a taboo

The workers interviewed understand the maintenance of life as the very intention of pre-hospital care.

*“It is because, within our routine, the issue of maintaining the patient’s life is very strong. In the case of palliative care that you are there just to give comfort, you are out of your routine, this causes you an emotional shock right away, because you will only give comfort, you will not fight for his life”* (25)*.*

Therefore, for the participants, death represents a taboo, a dilemma in their work in the PHC. It is possible to interpret in the testimonies a certain fear of health professionals towards the theme of death, as if death represented a failure of the professional’s performance.

*“It is because here in Brazil we have a very short view of death and to accept this fatality, which is something that we will all experience, right?… and the family that does not accept the disease, the state that the person is in, and they don’t even accept death, right?”* (3)

### Subtopic: professional training

This nucleus discusses the speeches of professionals who indicated the flaws in professional training regarding PC or even the lack of emotional preparation to deal with death. They also reflected about the curative education paradigm, in which professionals are trained to save lives at all costs.

*“No, I don’t feel prepared, my team doesn’t feel prepared for that either. It is an issue that must be developed, right? new studies, new protocols; because unfortunately we arrive, right? and we are used to arrive and do something to make a difference, doing what must be done.*” (10)

*“No, I don’t think so, by now. I was not prepared for this yet, we were prepared for, as you said yourself, right? to try to reestablish life, and not give this kind of news... this kind of news, no, right?! This kind of comfort.”* (16)

### Theme: decision making and ethical conflicts

This theme reflects the ethical conflicts reported by PHC professionals in decision making involving PC patients and their families during calls, as illustrated in the following report:

*“If this patient is really a terminal patient, this patient is not to progress any further, the family is aware of palliative care, but they cannot take the situation, the person’s suffering. So many times they tell us, please do it, I’m aware of it, they already explained it to me, but I want you to do something, I want you to invest, the family changes their opinion out of nothing … and then, what are we supposed to do? As long as I have a palliative care letter explaining everything correctly, indicated by the responsible doctor...”* (15)

The participants believed that the main conflicting aspects in their work was the family’s lack of knowledge regarding the patient’s will and the lack of access to the patient’s history (for PHC professionals). The existence of an advanced health care directive or living will could be of help in conflicts regarding decision making about invasive therapies that can prolong the patient’s suffering. In Brazil, there is still no legislation regulating the Anticipated Directives of Will (ADWs), only resolutions of the federal council of medicine, which still hinders actions aimed at the Anticipated Planning of Care and brings some insecurity in decision making during emergency care, in the absence of previous records of will, as indicated by the interviewed professional.

### Subtopic: family

The PHC professionals said that lack of prior clarification of family members about the clinical picture of palliative care and about the patient’s will is one of the greatest difficulties they face.

*“What happens is that, many times, no matter how much the family is informed, the family is not prepared, and they see that patient there in agony, unable to breathe and they end up calling us, and we have to be willing to help”* (14)

*“And today, as an emergency worker and rescuer, I think that we can alleviate the suffering as long as we know the history beforehand and that the family knows the history beforehand. Avoiding heroic measures to try to revive the patient in case of an eventual CRP, there would be no need for resuscitation if the family member were aware, if we knew that this patient is a palliative patient”* (23)

*“I doesn’t matter if I know that maybe that patient is in a terminal stage, but that the family does not accept that, I have to act as if the patient was not, because I don’t have any documents in the pre-hospital service that support me, telling me that I have to call the family and talk to them, no, let it be, this is the end of the person’s life, so that’s the biggest problem.”* (12)

### Subtopic: information and communication

The PHC professionals in this study attributed to the communication failures between health services and professionals the meaning of difficulties in decision making. They reported that information regarding the history, clinical condition, and reference services in PC for the referral of the patient is absent/limited.

*“Yes... as a doctor I have the obligation to take a quick action. If I am not aware that the patient is in palliative care, the family does not know that he is in palliative care, from that moment on this is a patient with prognosis. So, if he is a patient with a prognosis, then he is a patient with whom I will act as in an emergency, I will intubate, I will resuscitate, I will take all these measures”* (12)

*“For sure, for sure, and I bring again the issue of lack of information, so many times we make so many mistakes, the patient is many times in palliative care or not, and ends up going through invasive procedures, what it was maybe against the patient’s or the family’s desire, but we don’t have this information, there is nothing written”* (2)

*“And if we don’t have this history, if we don’t know or if we want to apply our protocol, which may go eventually against palliative care, it can certainly worsen or even influence the diagnosis or the survival of this palliative patient”* (10)

### Theme: Dysthanasia

Despite treating the subject of death as a taboo, the health professionals responding the study recognized that the professional performance in PHC contributes to an approach based on futile therapies that sometimes prolong the suffering of patients and family members.

The participants attributed to hospitalization and futile procedures the meaning of outcomes of this type of action at PHC.

### Subtopic: hospitalization

This sub-theme represents the professionals’ perceptions of the need to refer the patient to the hospital, even though they are aware of the implications in terms of prolonged suffering for the patient and family that hospitalization will cause.

*“But obviously if I arrive at a situation where the patient has respiratory failure and I take the attitude of intubating him or adopt an invasive action, I certainly prevent him from having a natural and good process of dying, what is actually expected for the process. Then he arrives at another service, intubated, then this becomes a snowball right?! Because he is intubated, then he needs to go to the ICU... so... the people who would die today, we have the means to keep the person alive and some cases for good and some other cases unfortunately for suffering... it increases the suffering, it prolongs the suffering... dysthanasia.”* (22)*.*

*“If the patient is giving his last breaths, I think he needs an access, oxygen, we need to take him to the hospital... he hasn’t died yet”* (19)*.*

### Subtopic: futile procedures

The professionals recognized futile procedures in PC patients during PHC, aspects that prolong suffering and lead to dysthanasia.

*“... unfortunately what we can do is get there, do some procedure and tell the family, unfortunately we cannot stay there at the patient’s residence, that we have to go away, and at the moment the patient stops breathing they will have to call us again to come and confirm, but I’ve already seen that some professionals are not prepared, they think that SAMU is only for Ahhhh it is an arrest, we have to resuscitate, let’s go... Oh no, we are going to do anything, so we’re not going to do anything. I don’t think that’s right.”* (14)*.*

*“...Then the relative comes there, please do something.... Obviously, if all of this is on record, documented, that he is a palliative that he has, okay, we will not do anything; if there is nothing written, and we do not have knowledge, it is our obligation to do the procedures... this is my opinion. Well, preparedness, when we talk about preparedness, we’re kind of.. well,.. like that, right?! I don’t know, I don’t know, I think I’m prepared to save lives.”* (15)*.*

## Discussion

The pre-hospital emergency service deals directly with saving lives and, therefore, faces ethical conflicts in decision making involving patients at risk of death. The distance from places with more resources, the provision of care at the patient’s residence, the realization of procedures on public roads, and a low number of team members contribute to make the work stressful and challenging. The work of these professionals with PC patients is even more challenging and conflicting because it involves conflicts related to futile therapies that prolong suffering.

The nuclei of meaning Death as taboo and Professional training emerging from the theme ‘unpreparedness’ represent the perceptions of the PHC professionals regarding the team’s inability to deal with death and care in the case of PC patients, which is reflected in the outcomes of care. Professionals identify death as a professional failure and understand that the role of the PHC service is to save lives, justifying the adoption of extraordinary procedures in the care of patients in PC.

Death is defined as a taboo to be deconstructed by all professional categories, because they understand death as a professional failure and not as an episode, that is part of life. Professionals often run away from terminal patients, do not talk about it, masking death [25].

Other authors [26] made an analysis of the professionals’ performance in face of their worldviews, considering the biomedical vision, where patients are seen as mere carriers of diseases and that the professional must be able to treat and cure at all costs and the holistic vision considering the integrality and factors that permeate health as the social and psychological. For professionals immersed in a biomedical vision, death is considered a professional failure that should be avoided at all costs.

Death anxiety has a high prevalence in some professional categories, among them the emergency medical team, due to occupational exposure to injured patients, as well as terminal patients, it can be seen through studies that found that the older the ES professional is, the lower his level and anxiety for death [27]. Other studies concluded that ES teams who reported personal experiences ‘resolved’ with grief were more comfortable with the end of resuscitation and patient death [28].

As understood by the participants in this study, professional training and continuing education need to address the issue of death, as observed in a study conducted in Canada. A research [29] analyzed the barriers for the implementation of PC in Canada in relation to four countries (United Kingdom, Ireland, New Zealand and Australia) and the results emphasized that significant investments in undergraduate and graduate courses in medicine and nursing are necessary to train professionals in PC, with knowledge and skills to meet the fundamental needs of this profile of patient. However, professionals who are already in the work market need continuing education to meet such demands.

It is a fact that it is necessary to reformulate the curricula in the training of health professionals, since there is a lack of disciplines involving the theme of death and palliative care, contributing to the debate on the subject and promoting greater security to the professional when faced with the issue of death [25].

The decision to suspend or terminate resuscitation is not easy and results suggest that the ES team, feel insufficiently prepared to communicate the death, as well as do not know how to deal with the anguish of the families, the researches point out the need for greater Interventions in professional training for the notification of death, emergency care, decision making of resuscitation and death of the patient, in order to better prepare the ES teams to deal with the various situations [30, 31]. One fact is that without adequate preparation and support, it seems that the team distances itself from good decision making in order to avoid the management of patient death and later involvement with family pain. Feeling unprepared and unsupported has been associated with increased anguish and anxiety regarding the death of health professionals [32, 33].

According to another study [34], members of the emergency team chose biomedical studies as more important when compared to the psychological and social approach. That said, the offer of additional training and continuing education aimed at death has an impact, favoring more reliable and assertive decisions, especially when associated with complex issues which are not part of the routine of professionals who deal with acute events.

The challenge for public policy managers, with the increase in demand for the care of PC by generalist professionals, leads one to think about what skills are needed to care for patients and their families. Communication skills, empathy, analysis of needs and preferences, anticipated care planning are required. These skills should be included in the curriculum of undergraduate and graduate health professionals. Even in the face of this insertion, there is no instrument that can be used to evaluate this competence in palliative care, because it is quite complex [35].

The theme ‘Decision making’ reflects ethical conflicts and their impact on care outcomes related to limited or even nonexistent information on the patient’s current health condition and the family approach at the moment of the occurrence.

The results of a qualitative research [36] revealed that professionals working in the pre-hospital service were concerned with the patient’s manifestations of will, but there was a possibility of contradicting the objectives of the service they perform. They also expressed a feeling of helplessness regarding the will of the family in relation to the NRO, and confessed that there was distrust in accepting the manifestations contained in the document even seeing the signature of the patient’s physician.

In order to assess the care provided to terminally ill patients in their homes, researchers [12] conducted a qualitative investigation in an English hospital and the results demonstrated that a set of factors impaired the ability of respondents to keep patients at their homes when they were close to the end of life. Such factors were: poor availability of support in the community, limited information about the patient and his health condition, and tendency towards invasive procedures in PHC.

The results found in the present study are consistent with the findings presented in that research. There is a need for quick decision making in PHC and the limitation of information about the patients’ history and care preferences often interferes with their permanence in their residence, leading to successive hospitalizations and the performance of futile procedures [12].

A systematic literature review [4] showed that in countries such as Australia, New Zealand, Canada and countries of Europe, where medical records of patients are shared by the health systems, the choice of actions and care measures in during emergency calls are facilitated. The study also emphasized that when the information is integrated between primary care and specialized PC services, positive factors are observed in terms of less repeated admissions and shortest hospital stay, besides relevant improvements in pain control and other symptoms and, consequently, improved Quality of Life (QOL) for patients.

The importance of a professional culture focused on Anticipated Care Planning, as a process of discussion and registration of patients’ preferences for future care and health decisions about the end of life, is also highlighted. A qualitative study conducted with health professionals from seven hospital PC teams in Brazil identified the need to approach the ADWs with the patient, emphasizing the importance of establishing bonds of trust with the patient, effective communication with the patient and the approach in the family context [37].

Thus, the lack of information on the current health status of the patient and the approach of the family at the time of the occurrence reflects the lack of a prior approach to ARVs with the patient and family, in addition to the lack of integration and communication between health services and professionals with the emergency and emergency services, as identified in the results of this study.

In addition, it becomes relevant to adopt an emergency care plan that allows doctors to discuss and record patient preferences in advance, not only in relation to CPR, but all aspects of care and treatment in an emergency, providing recommendations for care and treatment for future scenarios. A systematic review study has identified that these plans should be considered when attending people with complex health needs, limiting living conditions, or diseases that predispose to sudden deterioration or cardiac arrest. And that some triggers may indicate the need to initiate a discussion on emergency care plans, such as: requests from the patient himself; recognition of complex or long-term medical needs during care in clinics and wards of hospitals, as well as in general practice; diagnosis of a life-limiting condition – recognition that care at the end of life will be necessary; admission to a nursing home and identified risk of acute deterioration, cardiac arrest, or death [38].

The theme ‘dysthanasia’, which addressed the issue of carrying out futile procedures and referrals to hospital admissions, demonstrated the concern of professionals working in the emergency service with the actions adopted in the pre-hospital service in the country side of Brazil.

Despite this, the results of a study [39] demonstrated that it was more acceptable for the emergency team to start treatment and then leave it if the expected benefit was not obtained than “doing absolutely nothing” (p. 41). Death can be seen as a failure in the care provided and many professionals do not notice the value of choices and end-of-life desires, such as dying at home and in the presence of relatives [40].

It was observed in this study that, although the professionals understand that PC should be taken into account in cases assisted by the PHC, the lack of knowledge of family members and lack of integration between health services result in the implementation of futile therapies, which will only prolong the suffering of patients and families.

In this context, the curative medical model contributes considerably to disease-centered approaches, leading to the adoption of futile procedures for prolonging life, and a discouraging understanding about communication in terminal care between members of the multidisciplinary team, patients and family members. However, this culture can be modified directly through education and training, and can be also influenced by the success of previous cases, because positive experiences and results drive the team’s reasoning and encourage the continuous practice of actions [40].

Decisions involving whether or not to transport the patient to specialized services also generate conflicts within the PHC team, because keeping the patient at home involves decisions that go beyond clinical knowledge, and the rate of future calls for the emergency service is high when the patient remains at home [41].

In this context, Home Care (HC) is a type of assistance favorable to PC, since the philosophy and principles that guide PC and HC are similar. However, it is known that the curative, technical and biological aspects of care present in the health system offered in Brazil do not favor the implementation of these principles in the context of HC [42].

Other studies [43–45], indicate that, sometimes, the feeling of uncertainty and the fear of lawsuits come from limited and unreliable information regarding end-of-life patients. Thus, even when the team knows that transportation to the hospital will not benefit the patient’s current health condition, the professionals think that the stay in the home is not safe and end up making a decision that is often inappropriate or even contrary to the patient’s will.

Thus, if PHC professionals had information and established effective communication with other services, there would be discussions for better decisions about transportation, actions performed, and acceptance of the patient’s desires. In this context, the role of nurses in PHC stands out because they constitute the main link among professionals of the team and they have the ability to communicate effectively with patients and their families.

The limitations of this study include the small number of participants and the realization in the context of a single municipality; however, it is understood that the results of this research reflect the reality of PHC in Brazil.

## Conclusion

The perception of PHC professionals regarding the outcomes of the care provided to patients eligible for PC showed that the team felt unprepared when it comes to decision making involving PC patients. Such unpreparedness has to do with the view of death as a taboo and lack of training and continuing education on death and PC.

In the meantime, it is observed, based on the reports, that the professionals experienced ethical conflicts and barriers in decision making on a daily basis during PHC service. Such conflicts and barriers are due to the fact that despite the fact that there have been more discussions about PC and its guidelines in Brazil, the absence of a specific policy and lack of integration and communication between health services constitute obstacles for teams working in this kind of service.

Thus, it is concluded that there is a need for professional training that contemplates end-of-life issues associated with PC and its principles, such as: affirming life and considering death as a normal process not rushing or postponing death; integrating the psychological and spiritual aspects of patient and family care, including counseling on grief and improving the quality of life, as well as the development and implementation of a specific policy for PC that involves all levels of attention and that positions the PHC service in holistic patient care. Moreover, it becomes necessary the adoption of a unified information system that enables more effective behaviors that favor the will of patients without generating dissatisfaction of the team with relatives.

Moreover, nurses are components of the PHC teams who due to their greater contact with patients and families have a broad view of the patients’ social life and needs. They are essential to manage conflicts and act in the training of the team and to provide assistance when it comes to the spiritual and psychological suffering of patients and their families.

## Data Availability

The datasets generated and/or analyzed during the current study are not publicly available due the protection of participants’ identities but are available from the corresponding author on reasonable request.

## Abbreviations

ADWs: Anticipated Directives of Will
CPR: Cardiopulmonary resuscitation
COPD: Chronic Obstructive Pulmonary Disease
CRP: C-reactive protein
ES: Emergency Services
HC: Home Care
HCN: Health Care Network
HIV/AIDS: Acquired Human Immunodeficiency Syndrome
IAHPC: International Association for Hospice & Palliative Care
ICU: Intensive Care Unit
MOH: Ministry of Health
NHC: National Health Council
NRO: Non-Resuscitation Order
PC: Palliative Care
PHC: Pre-Hospital Care
PHC: Primary Health Care
QOL: Quality of Life
UEN: Urgency and Emergency Network
SAMU: Mobile Emergency Care Service
SUS: Unified Health System
UFMS: Federal University of Mato Grosso do Sul
WHO: World Health Organization
